# Behaviourally-informed household communications increase uptake of radon tests in a randomised controlled trial

**DOI:** 10.1038/s41598-023-47832-z

**Published:** 2023-11-21

**Authors:** Shane Timmons, Peter D. Lunn

**Affiliations:** 1 Economic and Social Research Institute, Whitaker Square, Sir John Rogerson’s Quay, Dublin, Ireland; 2 School of Psychology, Trinity College Dublin, Dublin, Ireland; 3 Department of Economics, Trinity College Dublin, Dublin, Ireland

**Keywords:** Human behaviour, Psychology, Natural hazards, Oncology, Psychology and behaviour

## Abstract

Exposure to radon gas is a leading cause of lung cancer. Testing homes for the gas is straightforward, yet most people do not undertake tests even when offered freely. We report a pre-registered randomised controlled trial of communications to encourage test uptake. Households (*N* = 3500) in areas at high risk of radon exposure were randomly assigned to receive (i) a the control letter from the national Environmental Protection Agency; (ii) a behaviourally-informed version of the control letter that incorporated multiple nudges, including reciprocity messages and numeric frequencies of risk; (iii) this same behaviourally-informed letter in a re-designed envelope; (iv) the behaviourally-informed letter in the re-designed enveloped with a radon risk map of the household’s county. The behaviourally-informed letter led to a large increase in test uptake, from 22% in the control condition to 33% (a 50% increase). There was no additional benefit of the re-designed envelope, which generated uptake of 30%. Including the map led some households to respond faster, but the overall uptake (26%) was weaker. The results have implications for public health communications with households and show the potential for techniques from behavioural science to help mitigate environmental risks.

Radon is a leading cause of lung cancer, but most people don’t know their level of exposure^1–3^. Radon gas is emitted from the earth’s crust and so is present in most countries^4^. Outdoors, it decays quickly and poses little risk. However, the gas can also seep into buildings through cracks in foundations or pipework and accumulate indoors, increasing the likelihood of inhalation and exposure of cells in the lungs to radioactive particles. As a result, radon causes over 80,000 global deaths per year with most exposure occurring in homes^5^. Exposure rates are estimated to be increasing due to modern building design (e.g., better insulation) and increased time at home post-pandemic^6,7^. Encouraging householders to test the level of radon in their home is hence a policy priority among public health officials and environmental protection agencies, yet testing rates remain stubbornly low even when kits are offered freely^8,9^. Voluntary surveys about radon risk perception and testing rates provides estimates that 7–10% households in the US, Canada and Europe have tested their home, although these may overestimate the true testing rate if those who have tested are more likely to respond to such surveys^10^. Our aim was to test whether informing communications with householders using behavioural science can increase test uptake.

From a psychological perspective, the contradiction between the health risks of radon and apathy towards home testing is perhaps unsurprising. There are no sensory cues that radon is accumulating; it is colourless, odourless, tasteless and leads to no immediately perceptible symptoms. Its effects are only experienced over long time periods and those affected by it may never realise they were exposed. These features likely exacerbate other biases that inhibit mitigative action. People are often overly optimistic about their risk of health complications^11^. Exposure usually occurs at homes, where most people expect health risks to be lower. Place familiarity is linked to reductions in perceived risk^12^. Hence radon’s attributes work against psychological mechanisms for perceiving and reacting to risk^13^.

As a result, most previous attempts to increase radon testing rates have had limited success^14^. Taking randomised controlled trials (RCTs) as the best evidence, few tests of ways to communicate with householders have observed significant increases in testing rates and none have generated rates above 20% among those who hadn’t already decided to order a test. Weinstein, Sandman and Roberts (1991) issued letters to householders and varied whether they contained general advice to test or specific information about high levels of radon in their area^15^. Results showed no difference in test kit orders. Weinstein, Lyon, Sandman and Cuite (1998) issued videos to householders that either emphasised the risks of radon, how easy it is to test for radon, or both^16^. Compared to a control group order rate of 5.1% among those who had not previously decided to test, 18.7% of those who received the combination video ordered a test kit. However, the authors note the length of the video and costs of production limit the scalability of the intervention. Another (non-randomised) evaluation of costly awareness campaigns showed positive effects, with approximately one quarter of households in participating areas having tested for radon versus less than 15% of those in non-participating areas^17^. Other trials that have shown positive effects have relied on rating-scale intentions rather than measuring behaviour^18^ or on small, non-representative samples^19,20^. Thus no trials to date have observed testing rates above 20% using low-cost, scalable interventions.

Our approach differs from previous trials and follows recent calls from experts to apply rigorous methods from social science to radon communications. The Potsdam Radon Communication Manifesto emphasises the need for science-based communication strategies that rely on theory and evidence in place of gut-feelings on what may work, for collaboration between policymakers and social scientists and the use of interactive risk maps to bolster personalised communications^14^. McLaughlin, Gutierrez-Villanueva and Perko (2022) stress the need to address deficits in radon risk communication as a priority for National Radon Control Strategies^21^. A recent systematic review of radon communication campaigns highlights substantial deficits in the methods used to evaluate campaigns, the need to pre-test communications and the absence of behaviourally-informed interventions (or “nudges”)^22,23^. We worked collaboratively with Ireland’s Environmental Protection Agency (EPA) to inform postal communications issued to householders using behavioural science and test these new communications via an RCT.

In our pre-registered trial, we selected a random sample of 3500 households from high radon areas in Ireland. For this study, a high radon area was defined as an area where the EPA predicts that more than 20% of domestic dwellings have radon concentrations above the national reference level of 200 Bq/m^3^. Households were randomised to receive either a control communication from the EPA to encourage household testing or one of three behaviourally-informed communications. The design was stepped, such that each treatment added an additional feature to the previous. The logic of using a stepped design rather than factorial design was to maximise statistical power.

Our first treatment was to apply a series of behavioural levers to letters that were scheduled for posting to households in high radon areas. Despite the now widespread use of “nudges” in applied public policy^24^, they have not yet been employed to encourage radon testing. Many individual nudges generate small effects^25^, but in combination can make meaningful impacts on policy goals (e.g. with saving account uptake^26^). Hence we opted to combine four nudges into one “behaviourally-informed” (BI) letter. First, informed by successful trials that have increased flu and COVID-19 vaccine uptake, we used wording that implied both ownership of a specific test kit and leveraged reciprocity by communicating to householders that a free radon test kit had been “reserved” for them^27,28^. Second, we communicated the risk of exposure using numeric frequencies (i.e., “1-in-5 homes have high levels of indoor radon gas”), following evidence that such communications increase intentions to test relative to simple statements of risk^29,30^. Third, we added a specific deadline for returning the order form in order to leverage urgency^31,32^. Fourth, we simplified the letter. Despite the addition of behavioural levers, we reduced the total letter wordcount by 20% and increased whitespace. Simplification is thought to increase the salience of the recommended action and has been shown to improve tax compliance ^33–35^.

For our second treatment, the BI letter was the same but it was issued in a re-designed envelope. Changing the design of envelopes is thought to increase their salience and hence the likelihood they will be opened and attended to. For example, one trial by UK energy regulator showed higher engagement with the retail energy market using supplier-branded envelopes compared to control ones^36^. Similarly, hand-written sticky notes on letters and envelopes have had large effects on survey participation rates^37,38^. Hence, we re-designed the EPA’s letter to include a digital version of a sticky note.

The third treatment was issued in the re-designed envelope, but the BI letter was further manipulated to include a “targeted” communication for the householder. Targeted risk communications are those aimed at population subgroups defined by shared characteristics^39^. We highlighted the distribution of radon risk in the householder’s county using a radon map. The logic here is that the county map would add a layer of personalisation to the letter, allowing householders to identify their home as residing in a high radon area on the map^40^. An online pre-test of the maps used showed they lead to a majority of people reporting a high willingness to test for radon^29^.

Our pre-registered hypothesis (https://osf.io/6bgxs) was that the BI letter would lead to a higher proportion of test kit orders than the control letter, with incremental increases in uptake for both the envelope and map. We further pre-registered exploratory tests by region and speed of response.

## Methods

The study was approved according to the Economic and Social Research Institute’s (ESRI) ethics policy and was carried out in accordance with relevant guidelines and regulations. The ESRI granted a waiver of consent, due to the risk of bias in response rates and hence the validity of the findings. The trial design and analysis plan were pre-registered at https://osf.io/6bgxs. Our sample size was determined through a power analysis to provide sufficient statistical power to detect a 4%-point increase in uptake of test kits, allowing for a non-delivery rate of 20% and assuming an uptake rate of 20% in the control condition (based on previous direct mails run by the EPA). Households (*N* = 3500) were randomly selected from a database of 598,166 households residing in High Radon Areas. The GeoDirectory, a commercially available database of Irish addresses, was used to generate the database of occupied domestic addresses in High Radon Areas. The sample were then randomly assigned to one of four treatment groups. Stata’s random number generator was used for both selection and assignment. A chi-square test for differences in the regional spread of households in each group was negative, χ^2^ (6) = 3.80, *p* = 0.704.

Copies of all letters are available at the project’s Open Science Framework page: https://osf.io/sxn75/. The “control” group (*n* = 875) received the letter designed for use by the EPA at the time of the study. The letter invited householders to carry out a free radon test. They were informed that their home had been chosen because they reside in a High Radon Area. The letter stated that the test was free of charge and emphasised that no one would call to their home. They were told to complete an enclosed form and return in a prepaid envelope to take part in the survey. Two pages of additional information on the EPA and radon were included. Previous communications using a similar structure had generated uptake rates of approximately 20%.

The “behaviourally informed” (BI) letter group (*n* = 875) emphasised that the regulator had “reserved a free radon test” the householder. They were informed they were “prioritised” because their home is in a High Radon Area, which was contextualised using natural frequency information (“1-in-5 homes have high levels of radon gas”). The letter emphasised that no one would call to their home and the call-to-action, to complete and return the included form by the specified date, was bolded. The word count was 20% lower than the control letter. The additional information was simplified to one page, with reference to remediation processes removed.

The “BI + envelope” group (*n* = 875) received the same simplified letter as the BI group but the letter was issued in a re-designed envelope. The envelope contained an image of a post-it with the writing “household selected for free radon test kit” in Lucida Handwriting font. The lip of the envelope also emphasised that the address had been selected for a free test kit.

The “BI + envelope + map” group (*n* = 875) received the simplified letter in the re-designed envelope, but a print-out of a radon map for the household’s county depicting the risk levels in different areas was also included. Householders were informed that they could see the estimated level of radon risk in their area on the enclosed map or by visiting the EPA’s website.

**Figure 1:**
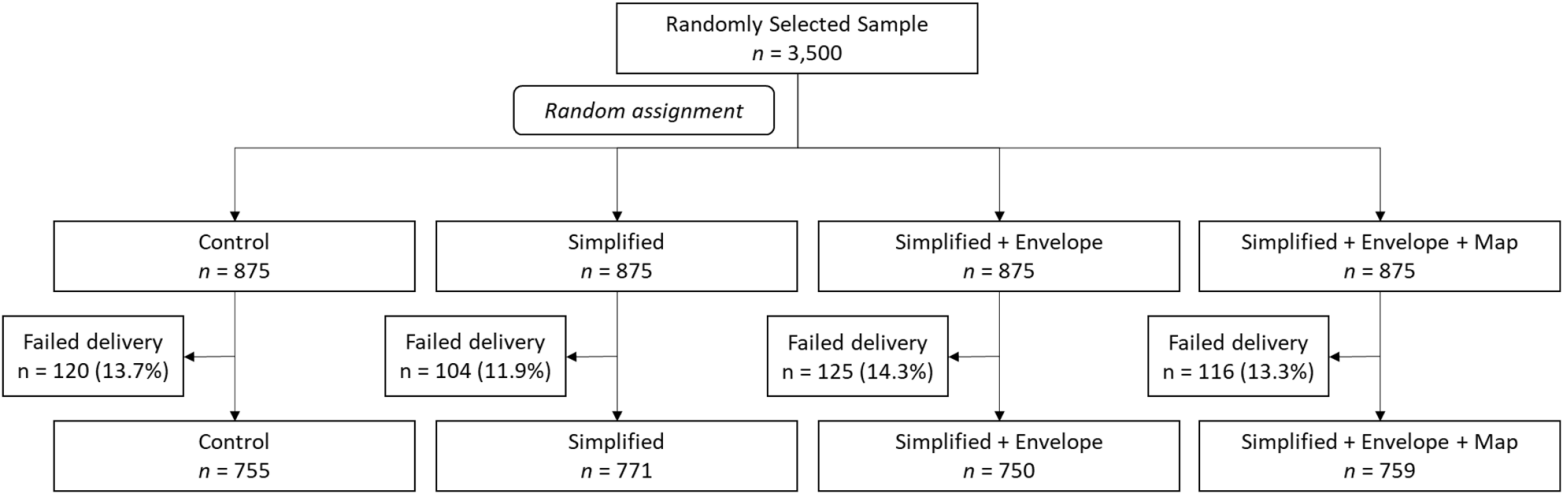
Flow diagram.

Letters were issued on March 9th and 10th 2023. The delivery failure rate was lower than expected at 13.3%. A chi-square test for differences in the delivery rate across groups was negative, χ^2^ (3) = 2.39, *p* = 0.496. Figure 1 tracks the process and presents final group totals. For successfully delivered letters, officers at the EPA recorded the date at which households responded and the mode of response (by returning the form or by phone) until April 12th. Just one household responded by phone, so we omit mode of response from analyses.

The final anonymised dataset included data on the letter issued by the household, their county, the date the letter was issued, if it delivered successfully, if the household responded, the date of any response and the response mode.

## Results

### Test uptake

**Figure 2:**
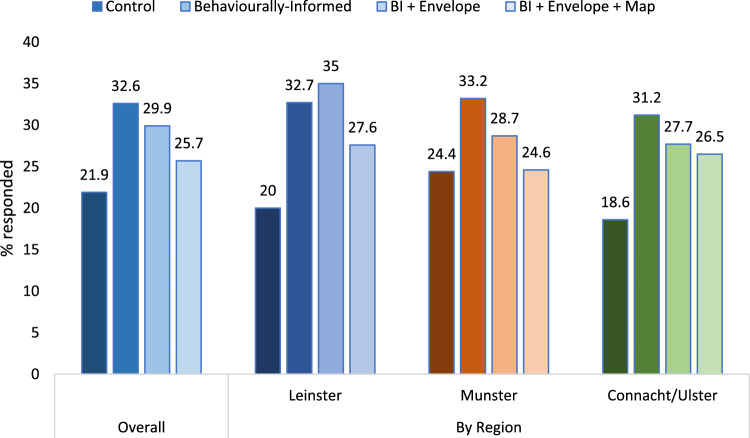
Uptake rate by treatment condition, overall and broken down by region.

The uptake rate among those with successfully delivered letters (*n* = 3035) was 27.5%. Figure 2 shows the overall uptake rate by treatment, which ranged from 21.9% in the control treatment to 32.6% among those who received only the BI letter. Table 1 presents a logistic regression predicting response from a categorical variable for treatment, controlling for household region. We use household region instead of county given interest in regional variation, but results are the same if county is used instead. We pre-registered including additional controls for date and mode of response, but all letters were issued at the same time and all responses were received via post. The model shows that all treatments led to a significantly higher uptake rate than the control letter. A test for equality of coefficients provides no evidence for a benefit of the redesigned envelope, for which the point-estimate is, in fact, lower (χ^2^ = 1.36, *p* = 0.242). The equivalent pairwise comparison of the BI letter in the redesigned envelope with the BI letter in the redesigned envelope with the map suggests that including the map marginally weakened the effect (χ^2^ = 3.13, *p* = 0.077). These effects are consistent if the model instead specifies dummy variables for each intervention (the BI letter, the redesigned envelope and the map), rather than the four-category treatment variable (reported in full in the Supplementary Online Material).

**Table 1:** Logistic regression model predicting uptake.

	Uptake
Treatment *(Ref: Control Letter)*	
BI Letter	0.55*** [0.32, 0.78]
BI Letter + Redesigned Envelope	0.42*** [0.18, 0.65]
BI Letter + Redesigned Envelope + Map	0.21* [− 0.02, 0.45]
Region *(Ref: Leinster)*	
Munster	− 0.06 [− 0.26, 0.15]
Connacht/Ulster	− 0.14 [− 0.38, 0.09]
Constant	− 1.21*** [− 1.44, − 0.98]
N	3035

*p < .10; **p < .05; ***p < .01. *P*-values for the treatment effects are one-tailed in line with the pre-registration. 95% confidence intervals are reported in brackets.

### Regional comparison

Figure 2 displays the uptake rates to each letter overall and in each of three regions. On average, 21.9% requested a test after receiving the control letter, 32.6% after receiving the simplified letter, 29.9% after receiving the simplified letter in the re-designed envelope and 25.7% when the county map was included. The pattern was similar across regions, with the BI letter alone performing better than all other treatments (except in Leinster, where the redesigned envelope produced a higher point estimate, but the difference is not statistically significant). Comparing the control group to the BI letter treatment, the effect size was much larger than predicted: a 49% (11%-point) increase in uptake rate.

### Time-to-respond

**Figure 3:**
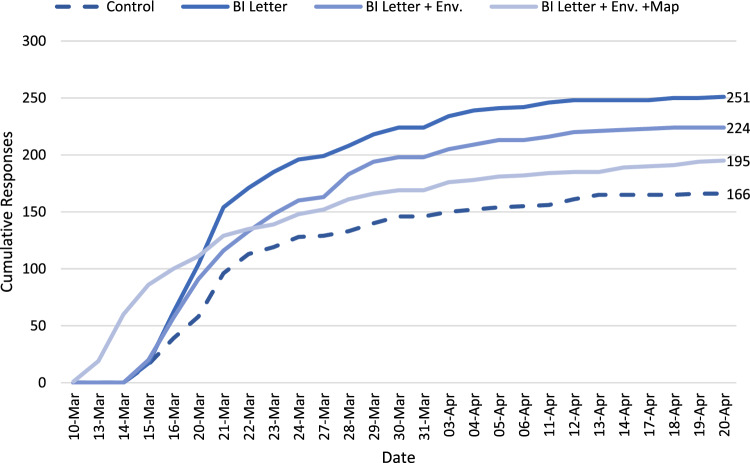
Cumulative responses by treatment condition over time. Note, the x-axis includes only business days, as responses were not recorded on weekends or public holidays.

We also tested time-to-respond to the letter using a survival model. A Cox regression (reported in the Online Supplementary Material) supports the OLS in Table 1. However, a test of the proportional hazards assumption for this model failed, χ^2^ (5) = 11.80, *p* = 0.038. Individual tests show that the failure was driven by the map intervention, χ^2^ (1) = 9.46, *p* = 0.002. This failure implies that the response ratio between those who received the map and those who didn’t was not constant over the trial period. Figure 3 presents the cumulative number of responses over the trial period and shows that the map treatment initially ordered tests more quickly than the other treatments (*Mdn* = 7 days vs. 11 days), despite a lower overall uptake rate.

## Discussion

Exposure to radon – and the associated risk of lung cancer – is increasing in multiple countries^7^. Household testing is hence a public health priority, but the lack of sensory cues from radon means most people don’t consider radon to be a risk^13^. Indeed, previous RCTs of direct communications with householders have failed to generate testing rates above 20%^16^. We therefore combined multiple behavioural interventions into one BI letter issued to householders in high radon areas.

The effect of combining interventions in this way was large. We observed an almost 50% (11%-point) increase in test uptake among those who received a BI letter, bringing the uptake rate to roughly 1-in-3. This effect is much larger than previous radon testing trials^16^ and the typical effect from interventions run by “Nudge Units”^25^. Although we can merely speculate, the size of the effect is perhaps driven by the combination of multiple behavioural levels into one intervention (e.g., reciprocity, numeric frequencies of risk, etc.) in a simple-to-read format, rather than relying on lengthy videos or singular levers. If all households in our 3500-home trial were issued our BI letter, 300 more homes in high radon areas would have ordered radon test kits over the trial period. The simplicity of the intervention – mere changes to a standard letter – means it is cheap to scale and is readily adaptable for testing in other countries.

While the strength of the effect for policymakers is encouraging, identifying the mechanism underlying it is not straightforward. The effect could have been driven by one feature (e.g., merely simplifying the text), two features (e.g., simplification and using numeric frequencies), three features, or all features (simplification, numeric frequencies, reciprocity, endowment and urgency). It’s also possible that one or more features had a negative effect, and the effect size of combining fewer features would have been even larger. Given the size of the five-feature effect relative to other interventions in the literature, however, we think this possibility is unlikely. Timmons et al. (2022) similarly observed large effect sizes from a ‘behavioural package’ interventions, relative to the standard approach of testing just one^26^. Hence, while our implications for psychological theory are limited, there is a clear benefit to combining multiple nudges into interventions that are driven by policy aims^41^.

That said, the results also point to the importance of individual tests of interventions that have greater costs (e.g., designing and printing new envelopes). We recorded no additional benefit of posting our BI letter in a redesigned envelope, despite the positive effects of such envelope manipulations in other studies^37^. The point estimate was in fact lower (albeit not statistically). One potential explanation for the lack of additional effect is the use of a digitalised sticky note with handwriting-style font rather than handwritten, physical one as used in the previous studies^42^. While speculative, this explanation implies that future tests of envelope redesign may benefit from preserving the physical component despite its additional cost. However, it is noteworthy here that published trials of envelope manipulations typically test the treatment effect when applied to issuing standard communications and not whether there is an *additional* benefit of envelope manipulations above behaviourally-informing the contents.

Moreover, while uptake rate remained 17% (4%-points) above the control letter when the printed map was included, this represents a reduction in uptake relative to the effectiveness of the BI letter alone. Other trials that have tested additional material with a letter have shown small positive effects on tax compliance (e.g., a flyer used by Hoy, McKenzie and Sinning, 2023^43^), but here too the effects are typically estimated relative to a standard communication and not against a behaviourally-informed letter. Hence, we again can merely speculate to explain why the map appears to have diminished the effect of changes to the letter alone. One possibility surrounds additional cognitive effort. Engaging with the map as well as reading the letter contents required householders to interpret the legend on the map and locate their home on the map. Those who exerted this effort may have been prompted to respond quickly, leading to a higher initial response rate, but those who initially avoided or put off expending the effort required may then have been less likely to return to the correspondence at a later stage.

It is worth dwelling on possible counterfactual experimental designs. Had we tested just one treatment that combined all interventions, we would have observed a relatively large effect, but for an intervention with greater resource costs for less benefit than merely adapting the letter. Note also that the response observation time had important implications for our assessment of the trial. Those who received the map responded significantly faster than other groups and, had we limited our observation period to just the first 2 weeks, we may have erroneously concluded superiority of this intervention.

Limitations of the trial present opportunities for future research. We recorded test uptake using a pre-existing system that required householders to return (via free post) forms to receive the test kits. Reducing frictions in the test kit ordering system, for example by implementing online applications, may lead to stronger effects. Moreover, there is scope to test interventions to improve downstream behaviours, such as test kit returns and, importantly, remediation rates among those who receive high radon readings as there is no health benefit associated with radon testing unless it leads to remediation. There is presently little research available to inform remediation behaviour trials^22,44^.

More broadly, our results demonstrate the benefit of using the psychology of risk perception and behavioural science to inform radon communications with householders. By doing so, we have demonstrated effective techniques for increasing testing rates for a substantial health hazard, thereby increasing the likelihood of householders remediating against the carcinogenic gas. The strength of the positive result we observe supports recent calls from experts for theoretically informed communications with householders that are evaluated using rigorous methods^14,21^. Further iterative testing of communications is required to help mitigate the negative health effects of radon.

### Supplementary Information

Supplementary Tables.

## Data Availability

Anonymised data are available on the project’s Open Science Framework page (https://osf.io/sxn75).
