# Supplementary material for: Behaviourally-informed household communications increase uptake of radon tests in a randomised controlled trial
Source: Sci Rep. 2023 Nov 21;13:20401. doi: 10.1038/s41598-023-47832-z (PMC10663451; doi:10.1038/s41598-023-47832-z)
Supplement: Supplementary file 1 — Supplementary Tables. [file 41598_2023_47832_MOESM1_ESM.docx]

Online Supplementary Material

for

Behaviourally-informed household communications increase uptake of radon tests in a randomised controlled trial

by

Shane Timmons & Peter D. Lunn

*Table A1. Logistic Regression Model Predicting Uptake (Alternative IV Coding)*

|  | Uptake |
| --- | --- |
| BI Letter  *(Ref: Control Letter)* | 0.55***  [0.32, 0.78] |
| Redesigned Envelope  *(Ref: Standard Envelope)* | -0.13  [-0.35, 0.09] |
| Risk Hazard Map  *(Ref: No Map)* | -0.20*  [-0.43, 0.02] |
| Region  *(Ref: Leinster)* |  |
| Munster | -0.06  [-0.26, 0.15] |
| Connacht/Ulster | -0.14  [-0.38, 0.09] |
| Constant | -1.21***  [-1.44, -0.98] |
| N | 3,035 |

**p* < .10; ***p <* .05; ****p* < .01. 95% confidence intervals are reported in brackets.

*Table A2. Cox Regression Model Predicting Uptake*

|  | Uptake |
| --- | --- |
| BI Letter  *(Ref: Control Letter)* | 0.46***  [0.27, 0.66] |
| Redesigned Envelope  *(Ref: Standard Envelope)* | -0.14  [-0.32, 0.04] |
| Risk Hazard Map  *(Ref: No Map)* | -0.12  [-0.31, 0.07] |
| Region  *(Ref: Leinster)* |  |
| Munster | -0.03  [-0.21, 0.13] |
| Connacht/Ulster | -0.10  [-0.30, 0.10] |
| N | 3,035 |

**p* < .10; ***p <* .05; ****p* < .01. 95% confidence intervals are reported in brackets.
